# Novel Tautomerisation Mechanisms of the Biologically Important Conformers of the Reverse Löwdin, Hoogsteen, and Reverse Hoogsteen G^*^·C^*^ DNA Base Pairs *via* Proton Transfer: A Quantum-Mechanical Survey

**DOI:** 10.3389/fchem.2019.00597

**Published:** 2019-09-18

**Authors:** Ol'ha O. Brovarets', Timothy A. Oliynyk, Dmytro M. Hovorun

**Affiliations:** ^1^Department of Molecular and Quantum Biophysics, Institute of Molecular Biology and Genetics, National Academy of Sciences of Ukraine, Kyiv, Ukraine; ^2^Department of Pharmacology, Bohomolets National Medical University, Kyiv, Ukraine; ^3^Department of Molecular Biotechnology and Bioinformatics, Institute of High Technologies, Taras Shevchenko National University of Kyiv, Kyiv, Ukraine; ^4^Department of Pathophysiology, Bohomolets National Medical University, Kyiv, Ukraine

**Keywords:** single proton transfer, double proton transfer, conformer, reverse Löwdin base pair, Hoogsteen, reverse Hoogsteen, transition state, quantum-mechanical calculation

## Abstract

For the first time, in this study with the use of QM/QTAIM methods we have exhaustively investigated the tautomerization of the biologically-important conformers of the G^*^·C^*^ DNA base pair—reverse Löwdin G^*^·C^*^(rWC), Hoogsteen G^*^′·C^*^(H), and reverse Hoogsteen G^*^′·C^*^(rH) DNA base pairs—*via* the single (SPT) or double (DPT) proton transfer along the neighboring intermolecular H-bonds. These tautomeric reactions finally lead to the formation of the novel G·${\text{C}}_{\text{O}2}^{*}$(rWC), ${\text{G}}_{\text{N}2}^{*}\cdot$C(rWC), G*′_N2_·C(rWC), ${\text{G}}_{\text{N}7}^{*}\cdot$C(H), and G*′_N7_·C(rH) DNA base mispairs. Gibbs free energies of activation for these reactions are within the range 3.64–31.65 kcal·mol^−1^ in vacuum under normal conditions. All TSs are planar structures (C_s_ symmetry) with a single exception—the essentially non-planar transition state TS_G*·C*(rWC)↔G^+^·C^−^(rWC)_ (C_1_ symmetry). Analysis of the kinetic parameters of the considered tautomerization reactions indicates that in reality only the reverse Hoogsteen G^*^′·C^*^(rH) base pair undergoes tautomerization. However, the population of its tautomerised state G*′_N7_·C(rH) amounts to an insignificant value−2.3·10^−17^. So, the G^*^·C^*^(rWC), G^*^′·C^*^(H), and G^*^′·C^*^(rH) base pairs possess a permanent tautomeric status, which does not depend on proton mobility along the neighboring H-bonds. The investigated tautomerization processes were analyzed in details by applying the author's unique methodology—sweeps of the main physical and chemical parameters along the intrinsic reaction coordinate (IRC). In general, the obtained data demonstrate the tautomeric mobility and diversity of the G^*^·C^*^ DNA base pair.

## Introduction

The study of the tautomerization mechanisms of the hydrogen (H) bonded nucleotide base pair is an important topic of modern quantum biophysics, biochemistry, molecular, and structural biology (Sinden, 1994; Sponer and Lankas, 2006; Alkorta et al., 2018). For over 65 years, this area of research has been under the intense scrutiny of both theoretics and experimentators, since the establishment of the spatial organization of DNA and formulation of the so-called “tautomeric hypothesis of the origin of spontaneous point mutations (transitions and transversions)” (Watson and Crick, 1953a,b; Erdmann et al., 2014) for this biologically important macromolecule—carrier of the genetic information, which is transmitted from generation to generation.

Lately, this tautomeric hypothesis has been experiencing an era of renaissance (Brovarets' and Hovorun, 2018). Thus, for the first time, within the framework of this hypothesis the new structural mechanisms of the tautomerization of pairs of nucleotide bases have been discovered, in which the transition of bases within the base pair into the mutagenic tautomeric form is accompanied by a significant change in the geometry of the base pair itself (Brovarets' and Hovorun, 2009, 2015a,b,c,d,e, 2016, 2018).

However, at the studying of the nature of the mutagenic tautomerization of DNA bases, the researchers limited themselves to the A·T and G·C Watson-Crick base pairs (Löwdin, 1963, 1966; Florian et al., 1994; Gorb et al., 2004; Bertran et al., 2006; Brovarets' and Hovorun, 2014a,b). Now this problem is considered more complex with the involvement of several biologically important conformers of these pairs (Hoogsteen, 1963; Pous et al., 2008; Alvey et al., 2014; Brovarets' and Hovorun, 2014a,b; Nikolova et al., 2014; Acosta-Reyes et al., 2015; Poltev et al., 2016; Zhou, 2016; Szabat and Kierzek, 2017; Ye et al., 2017).

These observations do not only allow to penetrate deeper into the essence of the phenomenon being studied, but also to answer, in particular, on a biologically important question—“Why *Nature* has exactly chosen the Watson-Crick DNA base pairs as elementary structural units for the construction of genetic material?”

Nowadays, there is an explicit bias to the A·T DNA base pair at the investigations of this type. This is due to a large number of circumstances, which will be outlined and discussed below.

Thus, it is widely known that the classical A·T(WC) Watson-Crick DNA base pair (Brovarets' and Hovorun, 2014b) may acquire different biologically significant conformations with various organization of the three intermolecular H-bonds—reverse Watson-Crick A·T(rWC), Hoogsteen A·T(H), and reverse Hoogsteen A·T(rH), which have been comprehensively studied in the literature (Hoogsteen, 1963; Pous et al., 2008; Alvey et al., 2014; Brovarets' and Hovorun, 2014b; Nikolova et al., 2014; Acosta-Reyes et al., 2015; Poltev et al., 2016; Zhou, 2016; Szabat and Kierzek, 2017; Ye et al., 2017).

Thus, in particular, in previous works (Hoogsteen, 1963; Pous et al., 2008; Brovarets' and Hovorun, 2010, 2014b, 2015f; Brovarets', 2013a,b; Alvey et al., 2014; Nikolova et al., 2014; Acosta-Reyes et al., 2015; Poltev et al., 2016; Zhou, 2016; Szabat and Kierzek, 2017; Ye et al., 2017; Brovarets' et al., 2018a,b,c,d,e,f; Brovarets' and Tsiupa, 2019) by the methods of quantum chemistry we have investigated in details the potential (electronic) energy surface of each of the four biologically important A·T DNA base pairs—Watson-Crick A·T(WC), reverse Watson-Crick A·T(rWC), Hoogsteen A·T(H) and reverse Hoogsteen A·T(rH), leading to the novel conformational or tautomeric states of these base pairs. It was theoretically demonstrated that these A·T(WC/rWC/H/rH) base pairs possess unique ability to perform conformationally-tautomeric transition into the planar wobble A^*^·T(w), A·${\text{T}}_{\text{O}2}^{*}$(w), and A·T^*^(w) base mispairs, non-planar wobble A·T(w_WC_), A·T(w_rWC_), A·T(w_H_), and A·T(w_rH_) base mispairs and incorrect A·T^*^(${\text{w}}_{\text{WC}}^{⊥}$), A·${\text{T}}_{\text{O}2}^{*}$(${\text{w}}_{\text{rWC}}^{⊥}$), A·T^*^(${\text{w}}_{\text{H}}^{⊥}$), and A·${\text{T}}_{\text{O}2}^{*}$(${\text{w}}_{\text{rH}}^{⊥}$) base mispairs containing mutagenic tautomers of the DNA bases, and also their interconversions between each other through the non-planar transition states *via* the structural or conformational rearrangements and intramolecular proton transfer along the intermolecular H-bonds.

In contrast to this, the classical G·C Watson-Crick DNA base pair (Brovarets', 2013b; Brovarets' and Hovorun, 2014a, 2015f) could not acquire different conformations in the main tautomeric state due to the obstacles presented by its geometrical structure. This, however, can be overcome through the G·C(WC) → G^*^·C^*^(WC) tautomerisation *via* the double proton transfer (DPT), according to Löwdin's mechanism (Löwdin, 1963, 1966; Brovarets' and Hovorun, 2014a, 2018). This Löwdin's reaction can proceed over the barrier of tautomerization or under the barrier *via* the tunneling (Parker and Van Everv, 1971; Boutis, 1992; Al-Khalili and McFadden, 2014; Brovarets' and Hovorun, 2015g; Godbeer et al., 2015; Turaeva and Brown-Kennerly, 2015; Meisner and Kastner, 2016; Roßbach and Ochsenfeld, 2017; McFadden and Al-Khalili, 2018; Pusuluk et al., 2018; Smedarchina et al., 2018; Shekaari and Jafari, 2019; Srivastava, 2019).

At this, the so-called Löwdin G^*^·C^*^(WC) base pair with geometry close to Watson-Crick, which is created in such a way, involving mutagenic tautomers of the DNA bases, can acquire similarly to the A·T(WC) base pair different conformations (Brovarets' and Hovorun, 2010, 2015f; Brovarets', 2013a,b; Brovarets' et al., 2018a,b,c,d,e,f; Brovarets' and Tsiupa, 2019)—reverse Löwdin G^*^·C^*^(rWC), Hoogsteen G^*^′·C^*^(H) and reverse Hoogsteen G^*^′·C^*^(rH) (Brovarets', 2013b) (here and below the superscript “^*^” denotes the rare tautomeric form of the DNA base (Glushenkov and Hovorun, 2016) and “′”—*trans*-orientation of the OH group). This demonstrates quite unexpected role of the Löwdin's tautomerisation for the conformational variety.

Currently, there is no mention of reverse Löwdin G^*^·C^*^(rWC), Hoogsteen G^*^′·C^*^(H) and reverse Hoogsteen G^*^′·C^*^(rH) conformers or their tautomerisation *via* the DPT along the intermolecular H-bonds, despite a great number of investigations devoted to this important phenomenon.

In our previous studies we have comperehensively investigated the tautomerisation *via* the DPT of the canonical A·T(WC) (Brovarets' and Hovorun, 2014b, 2015g) and G·C(WC) (Brovarets' and Hovorun, 2014a) Watson-Crick DNA base pairs, and also of the incorrect DNA base pairs—wobble G·T base pair (Brovarets' et al., 2015), short C·T (Brovarets' and Hovorun, 2013a), C^*^·C (Brovarets' and Hovorun, 2013b), T^*^·T (Brovarets' et al., 2014a), H·C (Brovarets' and Hovorun, 2013c; Brovarets' et al., 2013a) and H^*^·T (Brovarets' and Hovorun, 2013c; Brovarets' et al., 2013a); long A·A^*^ (Brovarets' and Hovorun, 2013d), A·G (Brovarets' et al., 2014b), G·G^*^ (Brovarets' and Hovorun, 2014c), H·H (Brovarets' and Hovorun, 2013c; Brovarets' et al., 2013a), H^*^·H (Brovarets' and Hovorun, 2013c; Brovarets' et al., 2013b) and H·A (Brovarets' and Hovorun, 2013c; Brovarets' et al., 2014c); Watson-Crick-like A·C^*^ (Brovarets' and Hovorun, 2015h), G^*^·T (Brovarets' and Hovorun, 2015i), G·A_syn_ (Brovarets' and Hovorun, 2014d), A^*^·${\text{G}}_{\text{syn}}^{*}$ (Brovarets' and Hovorun, 2014d), A^*^·A_syn_ (Brovarets' et al., 2014d), G·${\text{G}}_{\text{syn}}^{*}$ (Brovarets' and Hovorun, 2014e), T·2AP^*^(w) (Brovarets' et al., 2017; Brovarets' and Hovorun, 2019a), and G·2AP^*^(w) (Brovarets' et al., 2017; Brovarets' and Hovorun, 2019a) base mispairs and protein-DNA complexes (Brovarets' et al., 2012), which we have summarized in our review (Brovarets' and Hovorun, 2019b), devoted to the microstructural mechanisms of the tautomerization by the proton transfer along the neighboring intermolecular H-bonds in 22 biologically important pairs of nucleotide bases in the framework of the author's method, which enable to trace the evolution of the physico-chemical parameters along the intrinsic reaction coordinate (IRC).

In this study, we aim to reapply the approach, which we launched in our previous works (Brovarets' et al., 2018a,b,c,d,e,f; Brovarets' and Tsiupa, 2019) in order to investigate in details the tautomerisation of the reverse Löwdin G^*^·C^*^(rWC), Hoogsteen G^*^′·C^*^(H), and reverse Hoogsteen G^*^′·C^*^(rH) base pairs *via* PT along the neighboring intermolecular H-bonds.

As a result of the previous investigations, it was established that proton mobility along the intermolecular H-bonds does not change the tautomeric status of the investigated base pairs. Along with this biologically important conclusion, for the first time we have obtained a number of important physical and chemical characteristics. As such, it was documented that tautomerisation of the reverse Löwdin G^*^·C^*^(rWC) DNA base pair along the middle H-bond induces analogous SPT along its upper and lower H-bonds. Moreover, for the first time we have described the formation of a dynamically stable base pair with participation of the yilidic form of the purine base, formed through asynchronous DPT and participation of the CH group as proton donor.

Digging deeper into the mechanisms of tautomerisation of the reverse Löwdin G^*^·C^*^(rWC), Hoogsteen G^*^′·C^*^(H), and reverse Hoogsteen G^*^′·C^*^(rH) base pairs, we have carefully obtained sweeps of the physical and chemical parameters that characterize proton mobility along the IRC. Firstly, we have established a monotonic dependence of the base pair's dipole moment along the IRC. Second, it was shown that the SPT processes are characterized by the presence of 6 key points.

## Computational Methods

### Density Functional Theory Geometry and Vibrational Frequencies Calculations

All calculations of the geometries and harmonic vibrational frequencies of the considered base mispairs and transition states of their conversion have been conducted using Gaussian'09 package (Frisch et al., 2010) at the DFT B3LYP/6-311++G(d,p) level of theory (Lee et al., 1988; Parr and Yang, 1989; Tirado-Rives and Jorgensen, 2008), that has been already applied for analogous systems and approved to give accurate geometrical structures, normal mode frequencies, barrier heights, and characteristics of intermolecular H-bonds (Matta, 2010; Arabi and Matta, 2018; Gatti et al., 2018). We have used a scaling factor equal to 0.9668 in order to correct harmonic frequencies for the investigated base pairs (Brovarets' and Hovorun, 2010, 2015f; Brovarets', 2013a,b; Palafox, 2014; El-Sayed et al., 2015; Brovarets' et al., 2018a,b,c,d,e,f; Brovarets' and Tsiupa, 2019). We have associated structures, which were localized on the potential energy landscape by means of Synchronous Transit-guided Quasi-Newton method (Peng et al., 1996), to the minima or transition state (TS) by the absence or presence of the imaginary frequency in the vibrational spectra of the complexes, respectively. We used standard TS theory (Atkins, 1998) in order to estimate the forward and reverse barriers of the investigated tautomerisation reaction.

### IRC Calculations

Reaction pathways have been monitored by following IRC in the forward and reverse directions from each TS using Hessian-based predictor-corrector algorithm for integration (Hratchian and Schlegel, 2005). In such a way we ensure that it was received proper reaction pathway from reactants to products. Further, we have obtained the sweeps of the energetic, polar, and geometric characteristics of the H-bonds and base pairs along the IRC by calculating them at each point of the IRC (Brovarets' et al., 2017, 2018g,h).

### Single Point Energy Calculations

In order to take into account electronic correlation effects, we followed geometry optimizations with single point energy calculations using MP2 level of theory (Frisch et al., 1990) and 6-311++G(2df,pd) Pople's basis set of valence triple-ζ quality (Hariharan and Pople, 1973; Krishnan et al., 1980) and aug-cc-pVDZ Dunning's cc-type basis set (Kendall et al., 1992), augmented with polarization and/or diffuse function.

The Gibbs free energy G for all structures was calculated by the formula:

(1) $$\begin{array}{c} \text{G}={\text{E}}_{\text{el}}{\text{+E}}_{\text{corr}}, \end{array}$$

where E_el_–electronic energy, while E_corr_–thermal correction.

### Interaction Energies Calculations

We have obtained electronic interaction energies E_int_ at the MP2/6-311++G(2df,pd) level of theory as the difference between the total electronic energy of the base mispair and the electronic energies of the separate monomers. Gibbs free energy of interaction has been obtained using similar approach. At this, we also corrected the interaction energy for the basis set superposition error (BSSE) (Boys and Bernardi, 1970; Gutowski et al., 1986) according to the counterpoise procedure (Sordo et al., 1988; Sordo, 2001).

### Estimation of Kinetic Parameters

The time τ_99.9%_ spent for reaching the 99.9% of the equilibrium concentration between the initial and terminal base pairs in the system of reversible first-order forward (*k*_*f*_) and reverse (*k*_*r*_) reactions was estimated by formula (Atkins, 1998):

(2) $$\begin{array}{r} \tau_{99.9\%}=\frac{ln10^{3}}{k_{f}+k_{r}}, \end{array}$$

The lifetime τ of the base pairs was calculated using the formula 1/*k*_*r*_, where the values of the reverse *k*_*r*_ and forward *k*_*f*_ rate constants for the tautomerisation reactions were calculated as (Atkins, 1998):

(3) $$\begin{array}{r} k_{f,r}=\Gamma \cdot \frac{k_{B}T}{h}e^{-\frac{\Delta \Delta G_{f,r}}{RT}}, \end{array}$$

where quantum tunneling effects are accounted by Wigner's tunneling correction (Wigner, 1932; Brovarets' and Hovorun, 2014d), that has been successfully used for the DPT reactions (Brovarets' and Hovorun, 2013a,b,c,d, 2014c,d,e, 2015h,i; Brovarets' et al., 2013a,b, 2014a,b,c,d, 2015, 2017):

(4) $$\begin{array}{r} \Gamma =1+\frac{1}{24}{\left(\frac{h\nu_{i}}{k_{B}T}\right)}^{2}, \end{array}$$

where *k*_*B*_—Boltzmann's constant, *h*—Planck's constant, ΔΔ*G*_*f,r*_—Gibbs free energy for the forward (*f*) and reverse (*r*) tautomerisation reactions, ν_*i*_—value of the imaginary frequency at the TS of the tautomerisation reaction.

### QTAIM Analysis

Bader's quantum theory of Atoms in Molecules (QTAIM) was used to analyse the electron density distribution (Bader, 1990). The topology of the electron density was analyzed using program package AIMAll (Keith, 2010) with all default options and wave functions obtained at the level of theory used for geometry optimisation. The presence of the (3,−1) bond critical point (BCP), bond path between hydrogen donor and acceptor and positive value of the Laplacian at this BCP (Δρ >0) were considered altogether as criteria for the H-bond formation (Matta and Hernández-Trujillo, 2003; Matta et al., 2006a,b; Matta, 2014; Lecomte et al., 2015; Brovarets' and Pérez-Sánchez, 2016, 2017; Brovarets' et al., 2016).

### Energies of the Intermolecular H-Bonds

We calculated the energies of the intermolecular AH···B H-bonds in the base mispairs and TSs and of the sweeps of the H-bond energies by the empirical Espinosa-Molins-Lecomte (EML) formula (Espinosa et al., 1998; Matta et al., 2006b; Mata et al., 2011; Lecomte et al., 2015; Alkorta et al., 2016, 2017) based on the electron density distribution at the (3,−1) BCPs of the H-bonds:

(5) $$\begin{array}{r} E_{AH\cdot \cdot \cdot B}=0.5\cdot \text{V(r)}, \end{array}$$

where V(r)—value of a local potential energy at the (3,−1) BCP.

EML fomula has been also used for the estimation of the energy of the non-standard H-bonds CH···O in the stationary points of the base pairs on the hypersurface of their electronic energy.

We evaluated the energies of the classical NH···N/O and OH···O/N intermolecular AH···B H-bonds by the empirical Iogansen's formula (Iogansen, 1999):

(6) $$\begin{array}{r} E_{AH\ldots B}=0.33\;\sqrt{\Delta v-40}, \end{array}$$

where Δν–magnitude of the frequency shift of the stretching mode of the AH H-bonded group involved in the AH···B H-bond relatively the unbound group. We applied partial deuteration in order to minimize the effect of vibrational resonances (Brovarets' and Hovorun, 2014d,e, 2015h,i; Brovarets' et al., 2014d).

The energies of the NH···N and OH···O H-bonds in the TSs containing loosened covalent bridges were calculated by the Nikolaienko-Bulavin-Hovorun formula (Nikolaienko et al., 2012):

(7) $$\begin{array}{r} E_{NH\cdot \cdot \cdot N}=-2.03+225\cdot \rho , \end{array}$$

(8) $$\begin{array}{r} E_{OH\cdot \cdot \cdot O}=-3.09+239\cdot \rho , \end{array}$$

where ρ–the electron density at the (3,−1) BCP of the H-bond.

The atom numbering scheme for the DNA bases is conventional (Saenger, 1984).

## Results and Their Discussion

**Figure 1:**
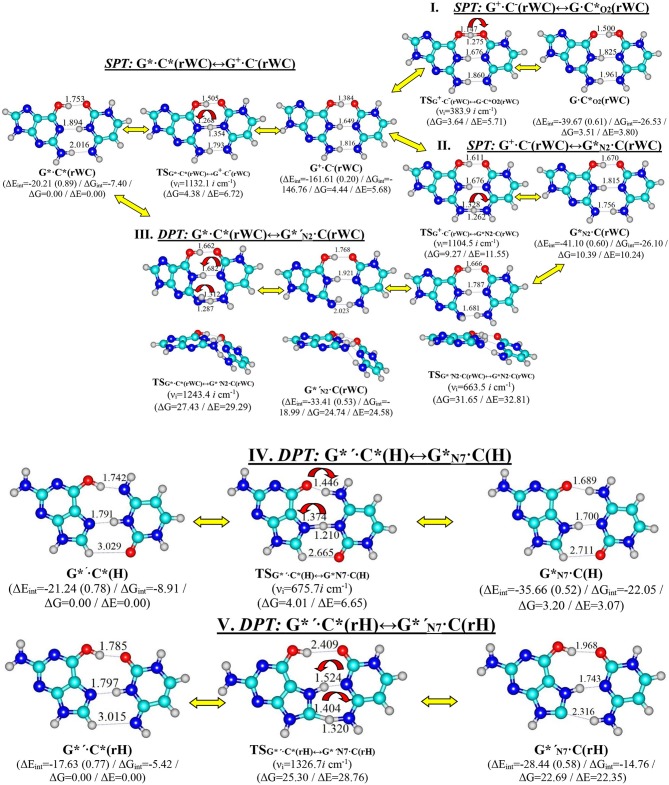
Discovered new reaction pathways of the tautomerization in the reverse Watson-Crick G*·C*(rWC) – I. G*·C*(rWC)↔G^+^·C^−^(rWC)↔G·C*_O2_(rWC), II. G*·C*(rWC)↔G^+^·C^−^(rWC)↔G*_N2_·C(rWC), III. G*·C*(rWC)↔G*′_N2_·C(rWC), Hoogsteen G*′·C*(H) – IV. G*′·C*(H)↔G*_N7_·C(H)—and reverse Hoogsteen G*′·C*(rH) – V. G*′·C*(rH)↔G*′_N7_·C(rH) – DNA base pairs through the single or double proton transfer. Electronic ΔE_int_ and Gibbs free ΔG_int_ energies of the interaction [MP2/6-311++G(2df,pd)//B3LYP/6-311++G(d,p) level of theory, in kcal·mol^−1^], relative Gibbs free energies ΔG, and electronic energies ΔE [MP2/aug-cc-pVDZ//B3LYP/6-311++G(d,p) level of theory in the continuum with ε = 1 at T = 298.15 in kcal·mol^−1^] are presented below complexes in brackets. Dotted lines indicate AH···B H-bonds – their lengths H···B are presented in angstroms; carbon atoms are in light-blue, nitrogen—in dark-blue, hydrogen—in gray and oxygen – in red. ν_*i*_—imaginary frequencies at the TSs of the tautomeric/conformational transitions.

In this paper we have investigated in details the tautomerisation processes *via* the single (SPT) or double (DPT) proton transfer of the G^*^·C^*^(rWC), G^*^′·C^*^(H), and G^*^′·C^*^(rH) base pairs along the neighboring intermolecular H-bonds as their intrinsically inherent property (Figure 1).

This paper is organized in the following way—firstly, we would discuss the tautomerisation process separately for each base pair and then we would present in details sweeps of the most important physico-chemical parameters along the IRC altogether for all investigated base pairs (Figures 1–12, Tables 1–8).

**Figure 2:**
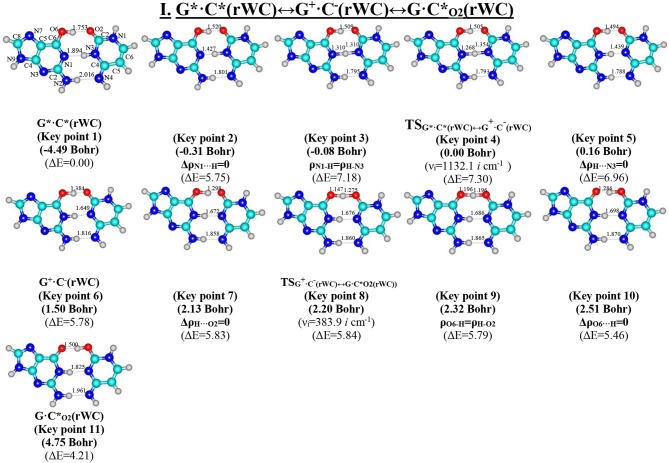
Geometric structures of the 11 key points describing the evolution of the I. G*·C*(rWC)↔G^+^·C^−^(rWC)↔G·C*_O2_(rWC) tautomerisation *via* the sequential SPT along the IRC obtained at the B3LYP/6-311++G(d,p) level of theory *in vacuo*. Coordinates of the 11 key points, their relative electronic energies ΔE (in kcal·mol^−1^ obtained at the B3LYP/6-311++G(d,p) level of theory in vacuum at T = 298.15) and imaginary frequencies ν_i_ (cm^−1^) at the TSs of their interconversions are presented below them in brackets (see Table 3). For more detailed designations see Figure 1.

**Figure 3:**
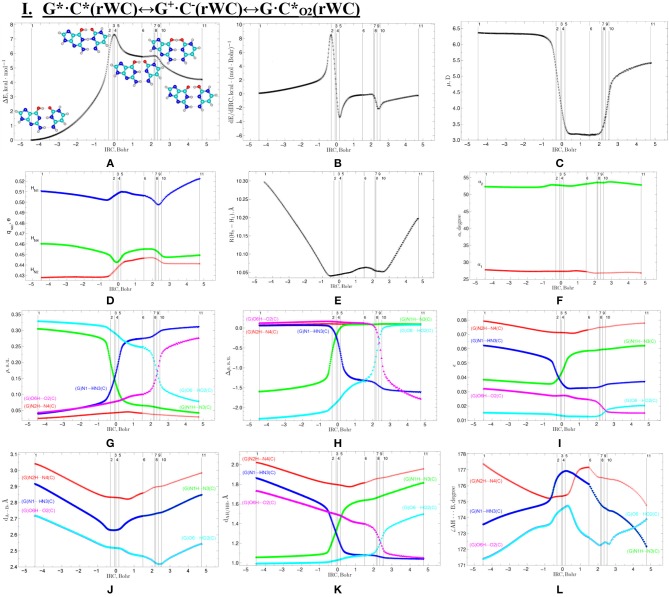
Profiles of: **(A)** the relative electronic energy ΔE, **(B)** the first derivative of the electronic energy with respect to the IRC (dE/dIRC), **(C)** the dipole moment μ, **(D)** the NBO charges q_NBO_, **(E)** the distance R(H_1_-H_9_) between the H_1_ and H_9_ glycosidic hydrogens, **(F)** the α_1_ (∠N1H_1_(C)H_9_(G)) and α_2_ (∠N9H_9_(G)H_1_(C)) glycosidic angles, **(G)** the electron density ρ; **(H)** the Laplacian of the electron density Δρ, **(I)** the ellipticity ε at the (3,−1) BCPs, **(J)** the distance d_A···*B*_ between the electronegative A and B atoms; **(K)** the distance d_AH/HB_ between the hydrogen and electronegative A or B atoms and **(L)** the angle ∠AH···B of the covalent and hydrogen bonds along the IRC of the investigated I. G*·C*(rWC)↔G^+^·C^−^(rWC)↔G·C*_O2_(rWC) tautomerisation *via* the sequential SPT obtained at the B3LYP/6-311++G(d,p) level of theory in vacuum.

**Figure 4:**
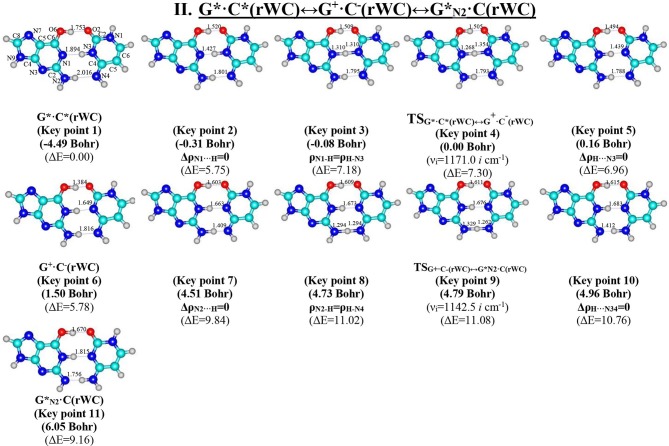
Geometric structures of the 11 key points describing the evolution of the II. G*·C*(rWC)↔G^+^·C^−^(rWC)↔G*_N2_·C(rWC) tautomerisation *via* the sequential SPT along the IRC obtained at the B3LYP/6-311++G(d,p) level of theory *in vacuo* (see Table 4). For more detailed designations see Figure 2.

**Figure 5:**
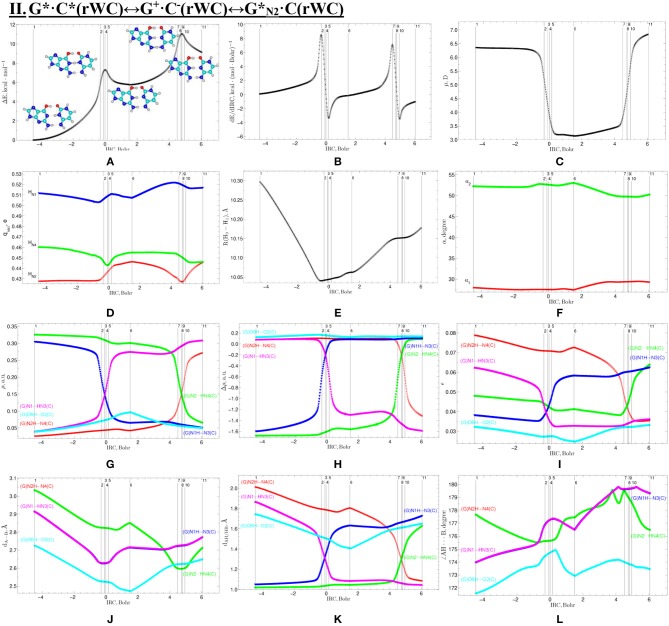
Profiles of physico-chemical parameters of the investigated II. G*·C*(rWC)↔G^+^·C^−^(rWC)↔G*_N2_·C(rWC) tautomerisation *via* the sequential SPT obtained at the B3LYP/6-311++G(d,p) level of theory in vacuum. For more detailed designations see Figure 3.

**Figure 6:**
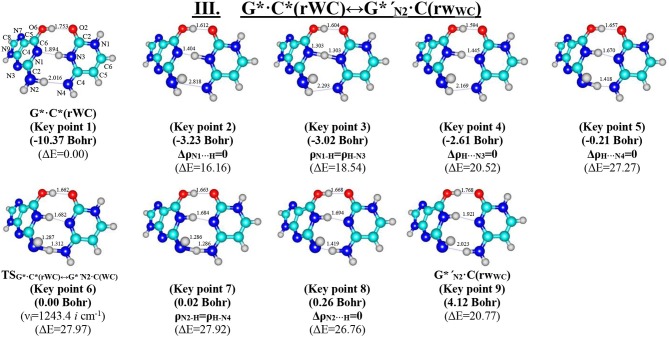
Geometric structures of the 9 key points describing the evolution of the III. G*·C*(rWC)↔G*′_N2_·C(rWC) tautomerisation *via* the DPT along the IRC obtained at the B3LYP/6-311++G(d,p) level of theory *in vacuo* (see Table 5). For more detailed designations see Figure 2.

**Figure 7:**
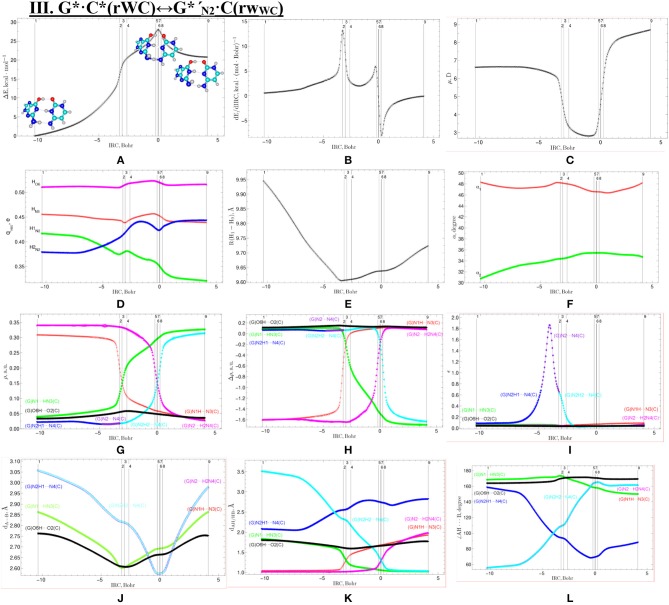
Profiles of the physico-chemical parameters of the investigated III. G*·C*(rWC)↔G*′_N2_·C(rWC) tautomerisation *via* the DPT obtained at the B3LYP/6-311++G(d,p) level of theory in vacuum. For more detailed designations see Figure 3.

**Figure 8:**
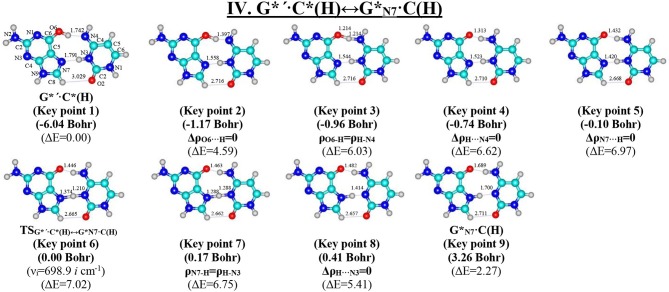
Geometric structures of the 9 key points describing the evolution of the IV. G*′·C*(H)↔G*_N7_·C(H) tautomerisation *via* the DPT along the IRC obtained at the B3LYP/6-311++G(d,p) level of theory *in vacuo* (see Table 6). For more detailed designations see Figure 2.

**Figure 9:**
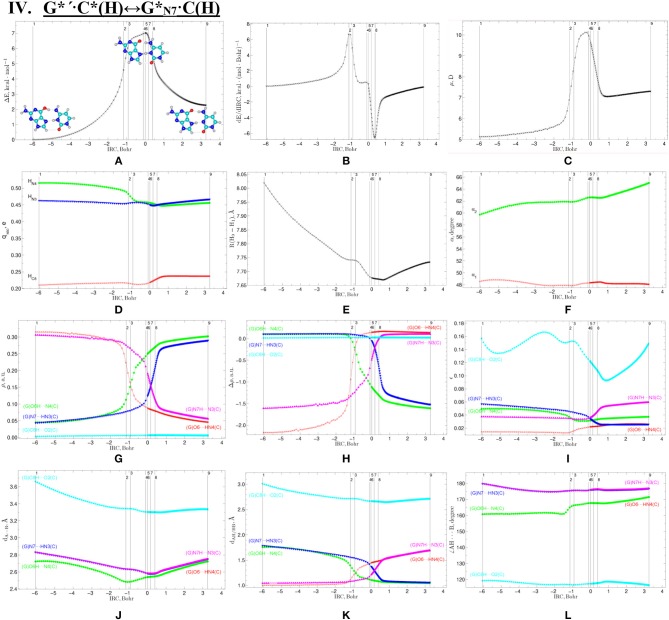
Profiles of the physico-chemical parameters of the investigated IV. G*′·C*(H)↔G*_N7_·C(H) tautomerisation *via* the DPT obtained at the B3LYP/6-311++G(d,p) level of theory in vacuum. For more detailed designations see Figure 3.

**Figure 10:**
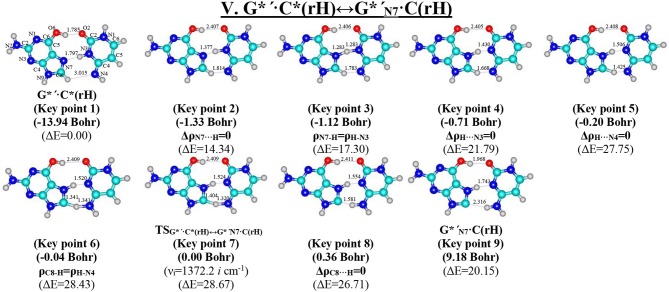
Geometric structures of the 9 key points describing the evolution of the V. G*′·C*(rH)↔G*′_N7_·C(rH) tautomerisation *via* the DPT along the IRC obtained at the B3LYP/6-311++G(d,p) level of theory *in vacuo* (see Table 7). For more detailed designations see Figure 2.

**Figure 11:**
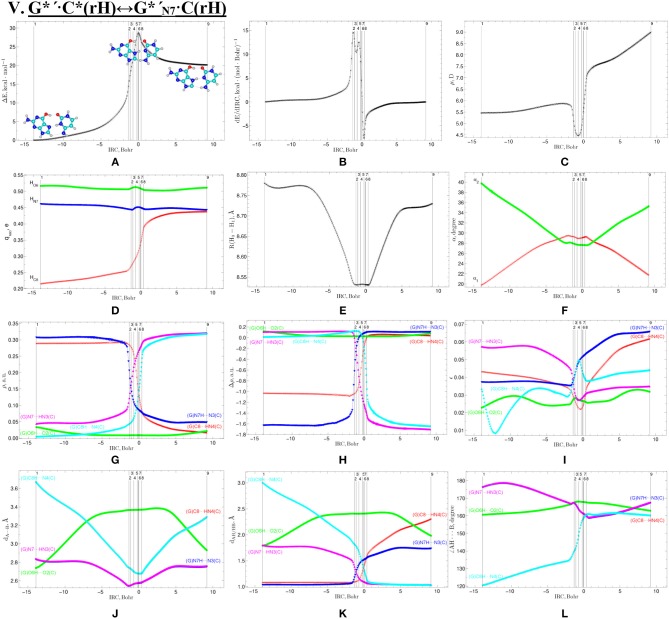
Profiles of the physico-chemical parameters of the investigated V. G*′·C*(rH)↔G*′_N7_·C(rH) tautomerisation *via* the DPT obtained at the B3LYP/6-311++G(d,p) level of theory in vacuum. For more detailed designations see Figure 3.

**Figure 12:**
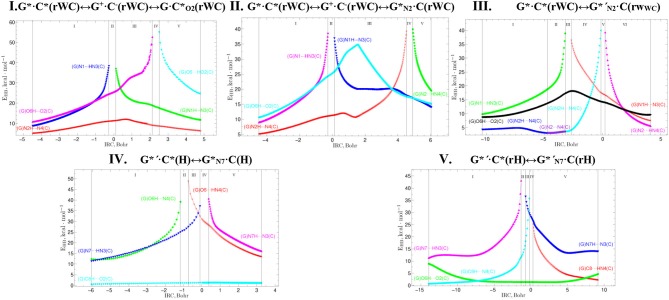
Profiles of the energy of the intermolecular H-bonds E_HB_ estimated by the EML formula at the (3,−1) BCPs along the IRC of the investigated tautomerisations *via* the SPT or DPT obtained at the B3LYP/6-311++G(d,p) level of theory (see Table 3).

**Table 1:** Energetic (in kcal·mol^−1^) and kinetic (in s) characteristics of the tautomerisation of the G^*^·C^*^(rWC), G^*^′·C^*^(H), and G^*^′·C^*^(rH) DNA base pairs *via* the SPT or DPT obtained at the MP2/aug-cc-pVDZ//B3LYP/6-311++G(d,p) level of QM theory in the continuum with ε = 1 under normal conditions (see Figure 1).

**Tautomeric transition**	**$\nu_{\text{i}}^{\text{a}}$**	**ΔG^b^**	**ΔE^c^**	**ΔΔ${\text{G}}_{\text{TS}}^{\text{d}}$**	**ΔΔ${\text{E}}_{\text{TS}}^{\text{e}}$**	**ΔΔG^f^**	****ΔΔ**E**^****g****^		**$\tau_{\text{99.9\%}}^{\text{h}}$**	****τ^i^****
							**kcal·mol^**−1**^**	**cm^**−1**^**		
G*·C*(rWC)↔G^+^·C^−^(rWC)	1132.1	4.44	5.68	4.38	6.72	−0.06	1.04	363.8	4.70·10^−13^	6.80·10^−14^
G^+^·C^−^(rWC)↔G·C*_O2_(rWC)	383.9	−0.93	−1.88	−0.80	0.03	0.13	1.91	668.1	2.10·10^−13^	1.77·10^−13^
G^+^·C^−^(rWC)↔G*_N2_·C(rWC)	1104.5	5.95	4.56	4.83	5.87	−1.12	1.30	454.7	7.96·10^−14^	1.15·10^−14^
G*·C*(rWC)↔G*′_N2_·C(rWC)	1243.4	24.74	24.58	27.43	29.29	2.69	4.71	1647.6	4.39·10^−11^	6.35·10^−12^
G*_N2_·C(rWC)↔G*′_N2_·C(rWC)	663.5	14.35	14.34	21.25	22.57	6.90	8.23	2878.9	9.24·10^−8^	1.34·10^−8^
G*′·C*(H)↔G*_N7_·C(H)	675.7	3.20	3.07	4.01	6.65	0.81	3.58	1252.3	3.07·10^−12^	4.46·10^−13^
G*′·C*(rH)↔G*′_N7_·C(rH)	1326.7	22.69	22.35	25.30	28.76	2.61	6.41	2242.2	3.56·10^−11^	5.15·10^−12^

a *The imaginary frequency at the TS of the tautomeric transition, cm^−1^*.

b *The Gibbs free energy of the initial relatively the terminal base pair of the tautomerisation reaction (T = 298.15 K)*.

c *The electronic energy of the initial relatively the terminal base pair of the tautomerisation reaction*.

d *The Gibbs free energy barrier for the forward tautomerisation reaction*.

e *The electronic energy barrier for the forward tautomerisation reaction*.

f *The Gibbs free energy barrier for the reverse tautomerisation reaction*.

g *The electronic energy barrier for the reverse tautomerisation reaction*.

h *The time necessary to reach 99.9% of the equilibrium concentration between the reactant and the product of the tautomerisation reaction*.

i *The lifetime of the product of the tautomerisation reaction*.

**Table 2:** Electron-topological, geometrical, and energetic characteristics of the intermolecular H-bonds in the investigated DNA base pairs in rare tautomeric forms and TSs of their tautomerization *via* the SPT or DPT obtained at the B3LYP/6-311++G(d,p) level of QM theory (ε = 1) (see Figure 1).

**Complex**	**AH···B H–bond**	**ρ^a^**	**Δρ^b^**	**100·ε^c^**	**${\text{d}}_{A\cdot \cdot \cdot B}^{\text{d}}$**	**${\text{d}}_{H\cdot \cdot \cdot B}^{\text{e}}$**	***∠AH*···*B*^f^**	***Δυ*^g^**	***EAH*···*B*^h^**	**μ^i^**
G*·C*(rWC)↔G^+^·C^−^(rWC)										
G*·C*(rWC) (Brovarets', 2013b)	O6H···O2	0.038	0.124	3.31	2.731	1.753	171.1	359.4	5.90	6.54
	N3H···N1	0.035	0.086	6.32	2.932	1.894	173.6	538.2	7.37	
	N2H···N4	0.026	0.077	8.04	3.036	2.016	178.0	248.9	4.77	
TS${}_{\text{G}*\cdot C*\left(rWC\right)↔G^{+}\cdot C^{-}\left(rWC\right)}$	O6H···O2	0.074	0.158	2.77	2.525	1.505	174.9	–	14.60**	3.92
	N2H···N4	0.043	0.102	7.06	2.829	1.793	175.2	–	7.64**	
G^+^·C^−^(rWC)	O6^+^H···O2^−^	0.103	0.113	2.40	2.458	1.384	172.4	1764.1	13.70	3.20
	N1^+^H···N3^−^	0.064	0.091	5.91	2.726	1.649	176.0	1092.3	10.70	
	N2^+^H···N4^−^	0.041	0.094	7.36	2.860	1.816	177.4	665.6	8.25	
I. G^+^·C^−^(rWC)↔G·C*_O2_(rWC)										
TS${}_{{\text{G}}^{+}\cdot C^{-}\left(rWC\right)↔G\cdot C*O2\left(rWC\right)}$	N1H···N3	0.059	0.097	5.98	2.740	1.676	174.6	–	11.01**	3.45
	N2H···N4	0.037	0.091	7.54	2.899	1.860	176.6	–	6.30**	
G·C*_O2_(rWC)	O2H···O6	0.074	0.150	2.00	2.532	1.500	173.7	1237.6	11.42	5.43
	N1H···N3	0.041	0.099	6.25	2.856	1.825	171.8	465.8	6.81	
	N2H···N4	0.029	0.082	7.83	2.987	1.961	174.7	368.5	5.98	
II. G^+^·C^−^(rWC)↔G*_N2_·C(rWC)										
TS${}_{{\text{G}}^{+}\cdot C^{-}\left(rWC\right)↔G*N2\cdot C\left(rWC\right)}$	O6H···O2	0.056	0.139	3.20	2.621	1.611	173.6	–	10.29**	4.99
	N1H···N3	0.059	0.103	6.10	2.733	1.676	179.9	–	11.25**	
G*_N2_·C(rWC)	O6H···O2	0.047	0.134	3.32	2.666	1.670	172.7	684.4	8.38	7.15
	N1H···N3	0.042	0.101	6.53	2.851	1.815	179.0	455.4	6.73	
	N4H···N2	0.048	0.094	7.08	2.816	1.756	176.4	846.1	9.37	
III. G*·C*(rWC)↔G*′_N2_·C(rWC)										
TS${}_{\text{G}*\cdot C*\left(rWC\right)↔G{*}^{\prime }N2\cdot C\left(rWC\right)}$	O6H···O2	0.049	0.131	3.84	2.663	1.662	170.6	–	10.29**	4.55
	N1H···N3	0.059	0.105	7.29	2.692	1.682	158.2	–	11.25**	
G*′_N2_·C(rWC)	O6H···O2	0.037	0.117	3.87	2.750	1.768	170.4	503.5	6.98	8.85
	N1H···N3	0.033	0.095	8.50	2.876	1.921	153.8	243.0	4.61	
	N4H···N2	0.024	0.071	11.36	3.014	2.023	160.2	409.3	6.22	
TS${}_{\text{G}*N2\cdot C\left(rWC\right)↔G{*}^{\prime }N2\cdot C\left(rWC\right)}$	O6H···O2	0.048	0.132	3.59	2.665	1.666	172.2	766.7	8.74	5.92
	N1H···N3	0.045	0.105	7.02	2.805	1.787	165.5	504.7	6.98	
	N4H···N2	0.058	0.082	5.23	2.759	1.681	173.4	1291.9	11.47	
IV. G*′·C*(H)↔G*_N7_·C(H)										
G*′·C*(H) (Brovarets', 2013b)	O6H···N4	0.047	0.102	4.88	2.710	1.742	160.7	691.6	8.42	5.16
	N3H···N7	0.032	0.101	5.68	2.834	1.791	180.0	572.6	7.62	
	C8H···O2	0.003	0.012	1.73	3.675	3.029	118.9	−4.1	0.55*	
TS${}_{\text{G}{*}^{\prime }\cdot C*\left(H\right)↔G*N7\cdot C\left(H\right)}$	N4H···O6	0.088	0.143	2.19	2.539	1.446	167.7	–	17.77**	9.63
	C8H···O2	0.007	0.024	12.24	3.303	2.665	117.4	–	1.27*	
G*_N7_·C(H)	N4H···O6	0.045	0.137	2.53	2.728	1.689	172.2	13.2	8.03	7.32
	N7H···N3	0.054	0.099	5.95	2.758	1.700	177.2	15.5	9.26	
	C8H···O2	0.006	0.022	16.54	3.324	2.711	115.8	1.2	1.15*	
V. G*′·C*(rH)↔G*′_N7_·C(rH)										
G*′·C*(rH) (Brovarets', 2013b)	O6H···O2	0.034	0.120	2.25	2.733	1.785	160.6	319.0	5.51	5.43
	N3H···N7	0.042	0.101	2.73	2.838	1.797	176.3	535.6	7.35	
	C8H···N4	0.004	0.013	3.58	3.681	3.015	120.4	−2.1	0.63*	
TS${}_{\text{G}{*}^{\prime }\cdot C*\left(rH\right)↔G{*}^{\prime }N7\cdot C\left(rH\right)}$	O6H···O2	0.009	0.027	2.66	3.368	2.409	167.9	–	1.36**	5.52
	N7H···N3	0.086	0.087	5.18	2.576	1.524	161.1	–	17.32**	
G*′_N7_·C(rH)	O6H···O2	0.022	0.080	3.15	2.918	1.968	162.9	112.6	2.81	9.01
	N7H···N3	0.049	0.114	6.60	2.758	1.743	167.8	619.6	7.94	
	N4H···C8^−^	0.016	0.038	6.19	3.302	2.316	160.1	339.7	5.71	

a *The electron density at the (3,−1) BCP of the H-bond, a.u*.

b *The Laplacian of the electron density at the (3,−1) BCP of the H-bond, a.u*.

c *The ellipticity at the (3,−1) BCP of the H-bond*.

d *The distance between the A and B atoms of the AH···B H-bond, Å*.

e *The distance between the H and B atoms of the AH···B H-bond, Å*.

f *The H-bond angle, degree*.

g *The redshift of the stretching vibrational mode υ(AH) of the AH H-bonded group, cm^−1^*.

h *Energy of the H-bonds, calculated by Iogansen's (Iogansen, 1999), Espinose-Molins-Lecomte (Espinosa et al., 1998; Matta et al., 2006b; Mata et al., 2011; Lecomte et al., 2015; Alkorta et al., 2016, 2017) (marked with an asterisk), or Nikolaienko-Bulavin-Hovorun (Nikolaienko et al., 2012) (marked with a double asterisk) formulas, kcal·mol^−1^*.

i *The dipole moment of the complex, D*.

**Table 3:** Electron-topological and structural characteristics of the specific intermolecular bonds revealed in the 11 key points and the polarity of the latters along the IRC of the I. G*·C*(rWC)↔G^+^·C^−^(rWC)↔G·C*_O2_(rWC) tautomerisation obtained at the B3LYP/6-311++G(d,p) level of theory in vacuum (see Figure 2).

**Complex**	**AH···B H-bond/ A-H/H-B covalent bond**	***ρ*^a^**	***Δρ*^b^**	***100·ε*^c^**	***${\text{d}}_{A\cdot \cdot \cdot B}^{\text{d}}$***	***${\text{d}}_{H\cdot \cdot \cdot B}^{\text{e}}$***	**_***∠AH*···*B***_^f^**	**μ^g^**
Key point 1 (−4.49 Bohr): G*·C*(rWC)	O6H···O2	0.038	0.124	3.31	2.731	1.753	171.1	6.54
	N1···HN3	0.035	0.086	6.32	2.932	1.894	173.6	
	N2H···N4	0.026	0.077	8.04	3.036	2.016	178.0	
Key point 2 (−0.31 Bohr): Δρ_N1···*H*_ = 0	O6H···O2	0.070	0.166	2.75	2.528	1.520	174.6	5.19
	N1···HN3	0.110	0.000	4.31	2.621	1.427	176.7	
	N2H···N4	0.042	0.105	7.07	2.831	1.801	175.2	
Key point 3 (−0.08 Bohr): ρ_N1−H_ = ρ_H−N3_	O6H···O2	0.073	0.160	2.76	2.526	1.509	174.9	4.23
	N1-H/H-N3	0.148	−0.193	3.73	2.620	1.310	177.0	
	N2H···N4	0.043	0.103	7.06	2.829	1.795	175.2	
Key point 4 (0.00 Bohr): TS_G*·C*(*rWC*)↔*G*+·*C*−(*rWC*)_	O6H···O2	0.074	0.158	2.76	2.525	1.505	174.9	3.92
	N1-HN3	0.166	−0.306	3.58	2.621	1.268	177.1	
	N1H-N3	0.133	−0.111	4.28	2.621	1.354	177.1	
	N2H···N4	0.043	0.102	7.06	2.829	1.793	175.2	
Key point 5 (0.16 Bohr): Δρ_H···*N*3_ = 0	O6H···O2	0.076	0.152	2.77	2.523	1.494	175.0	3.45
	N1H···N3	0.107	0.004	4.75	2.625	1.439	177.1	
	N2H···N4	0.044	0.100	7.05	2.827	1.788	175.3	
Key point 6 (1.50 Bohr): G^+^·C^−^(rWC)	O6H···O2	0.096	0.126	2.50	2.459	1.384	173.1	3.15
	N1H···N3	0.067	0.089	5.79	2.731	1.649	176.3	
	N2H···N4	0.042	0.095	7.24	2.861	1.816	177.0	
Key point 7 (2.13 Bohr): Δρ_H···*O*2_ = 0	O6H···O2	0.130	0.006	2.13	2.421	1.298	171.9	3.35
	N1H···N3	0.060	0.096	5.97	2.738	1.672	174.7	
	N2H···N4	0.037	0.091	7.53	2.897	1.858	176.6	
Key point 8 (2.20 Bohr): TS${}_{{\text{G}}^{+}\cdot C^{-}\left(rWC\right)↔G\cdot C*O2\left(rWC\right)}$	O6H-O2	0.138	−0.045	2.05	2.416	1.275	171.9	3.45
	O6-HO2	0.199	−0.644	1.34	2.416	1.147	171.9	
	N1H···N3	0.059	0.097	5.98	2.740	1.676	174.6	
	N2H···N4	0.037	0.091	7.54	2.899	1.860	176.6	
Key point 9 (2.32 Bohr): ρ_O6−H_ = ρ_H−O2_	O6-H/H-O2	0.172	−0.334	1.43	2.409	1.196	172.0	3.77
	N1H···N3	0.058	0.100	6.00	2.743	1.686	174.4	
	N2H···N4	0.037	0.091	7.56	2.902	1.865	176.6	
Key point 10 (2.51 Bohr): Δρ_O6···*H*_ = 0	O6···HO2	0.134	−0.006	1.63	2.411	1.286	172.0	4.48
	N1H···N3	0.056	0.104	6.01	2.747	1.698	174.3	
	N2H···N4	0.036	0.092	7.57	2.904	1.870	176.6	
Key point 11 (4.75 Bohr): G·C*_O2_(rWC)	O6···HO2	0.074	0.150	2.00	2.532	1.500	173.7	5.43
	N1H···N3	0.041	0.099	6.25	2.856	1.825	171.8	
	N2H···N4	0.029	0.082	7.83	2.987	1.961	174.7	

a *The electron density at the (3,−1) BCP, a.u*.

b *The Laplacian of the electron density at the (3,−1) BCP, a.u*.

c *The ellipticity at the (3–1) BCP*.

d *The distance between the A (H-bond donor) and B (H-bond acceptor) atoms of the H-bonds, Å*.

e *The distance between the H and B atoms of the H-bonds, Å*.

f *The H-bond angle, degree*.

g *The dipole moment of the complex, D*.

**Table 4:** Electron-topological and structural characteristics of the specific intermolecular bonds revealed in the 11 key points and the polarity of the latters along the IRC of the II. G*·C*(rWC)↔G^+^·C^−^(rWC)↔G*_N2_·C(rWC) tautomerisation obtained at the B3LYP/6-311++G(d,p) level of theory in vacuum (see Figure 4).

**Complex**	**AH···B H-bond/A-H/H-B covalent bond**	***ρ*^a^**	***Δρ*^b^**	***100·ε*^c^**	***${\text{d}}_{A\cdot \cdot \cdot B}^{\text{d}}$***	***${\text{d}}_{H\cdot \cdot \cdot B}^{\text{e}}$***	**_***∠AH*···*B***_*^**f**^***	**μ^g^**
Key point 1 (−4.49 Bohr): G*·C*(rWC)	O6H···O2	0.038	0.124	3.31	2.731	1.753	171.1	6.54
	N1···HN3	0.035	0.086	6.32	2.932	1.894	173.6	
	N2H···N4	0.026	0.077	8.04	3.036	2.016	178.0	
Key point 2 (−0.31 Bohr): Δρ_N1···*H*_ = 0	O6H···O2	0.070	0.166	2.75	2.528	1.520	174.6	5.19
	N1···HN3	0.110	0.000	4.31	2.621	1.427	176.7	
	N2H···N4	0.042	0.105	7.07	2.831	1.801	175.2	
Key point 3 (−0.08 Bohr): ρ_N1−H_ = ρ_H−N3_	O6H···O2	0.073	0.160	2.76	2.526	1.509	174.9	4.23
	N1-H/H-N3	0.148	−0.193	3.73	2.620	1.310	177.0	
	N2H···N4	0.043	0.103	7.06	2.829	1.795	175.2	
Key point 4 (0.00 Bohr): TS_G*·C*(*rWC*)↔*G*+·*C*−(*rWC*)_	O6H···O2	0.074	0.158	2.76	2.525	1.505	174.9	3.92
	N1-HN3	0.166	−0.306	3.58	2.621	1.268	177.1	
	N1H-N3	0.133	−0.111	4.28	2.621	1.354	177.1	
	N2H···N4	0.043	0.102	7.06	2.829	1.793	175.2	
Key point 5 (0.16 Bohr): Δρ_H···*N*3_ = 0	O6H···O2	0.076	0.152	2.77	2.523	1.494	175.0	3.45
	N1H···N3	0.107	0.004	4.75	2.625	1.439	177.1	
	N2H···N4	0.044	0.100	7.05	2.827	1.788	175.3	
Key point 6 (1.50 Bohr): G^+^·C^−^(rWC)	O6H···O2	0.096	0.126	2.50	2.449	1.384	173.1	3.15
	N1H···N3	0.067	0.089	5.79	2.731	1.649	176.3	
	N2H···N4	0.042	0.095	7.24	2.861	1.814	177.0	
Key point 7 (4.51 Bohr): Δρ_N2···*H*_ = 0	O6H···O2	0.057	0.136	3.19	2.620	1.603	173.8	3.91
	N1H···N3	0.061	0.096	6.06	2.731	1.663	179.8	
	N2H···N4	0.112	−0.001	4.86	2.592	1.409	180.0	
Key point 8 (4.73 Bohr): ρ_N2−H_ = ρ_H−N4_	O6H···O2	0.056	0.138	3.20	2.621	1.609	173.7	4.72
	N1H···N3	0.059	0.102	6.09	2.733	1.673	179.9	
	N2-H/H-N4	0.152	−0.211	4.15	2.590	1.294	179.4	
Key point 9 (4.79 Bohr): TS_G+·*C*−(*rWC*)↔G*N2·*C*(*rWC*)_	O6H···O2	0.056	0.139	3.21	2.621	1.611	173.6	4.99
	N1H···N3	0.059	0.103	6.10	2.733	1.676	179.9	
	N2-HN4	0.139	−0.130	4.59	2.591	1.329	179.3	
	N2H-N4	0.165	−0.300	4.01	2.591	1.262	179.3	
Key point 10 (4.96 Bohr): Δρ_H···*N*4_ = 0	O6H···O2	0.055	0.140	3.21	2.622	1.615	173.6	5.74
	N1H···N3	0.058	0.106	6.12	2.735	1.683	179.9	
	N2···HN4	0.111	0.000	5.10	2.594	1.412	179.0	
Key point 11 (6.05 Bohr): G*_N2_·C(rWC)	O6H···O2	0.047	0.134	3.32	2.666	1.670	172.7	7.15
	N1H···N3	0.042	0.101	6.53	2.851	1.815	179.0	
	N2···HN4	0.048	0.094	7.08	2.816	1.756	176.4	

*For footnote definitions see Table 3*.

**Table 5:** Electron-topological and structural characteristics of the specific intermolecular bonds revealed in the 9 key points and the polarity of the latters along the IRC of the III. G*·C*(rWC)↔G*′_N2_·C(rWC) tautomerisation obtained at the B3LYP/6-311++G(d,p) level of theory in vacuum (see Figure 6).

**Complex**	**AH···B H-bond/ A-H/H-B covalent bond**	***ρ^a^***	***Δρ*^b^**	***100·ε^c^***	***${\text{d}}_{A\cdot \cdot \cdot B}^{\text{d}}$***	***${\text{d}}_{H\cdot \cdot \cdot B}^{\text{e}}$***	**_***∠AH*···*B***_*^**f**^***	**μ^g^**
Key point 1 (−10.37 Bohr): G*·C*(rWC)	O6H···O2	0.038	0.124	3.31	2.731	1.753	171.1	6.54
	N1···HN3	0.035	0.086	6.32	2.932	1.894	173.6	
	N2H···N4	0.026	0.077	8.04	3.036	2.016	178.0	
Key point 2 (−3.23 Bohr): Δρ_N1···*H*_ = 0	O6H···O2	0.055	0.147	3.48	2.608	1.612	169.8	4.99
	N1···HN3	0.115	−0.010	4.65	2.602	1.404	173.1	
	N2···N4	0.018	0.065	65.91	2.818	–	–	
Key point 3 (−3.02 Bohr): ρ_N1−H_ = ρ_H−N3_	O6H···O2	0.057	0.144	3.48	2.607	1.604	170.3	4.17
	N1-H/H-N3	0.150	−0.187	4.06	2.603	1.303	172.4	
	N2H···N4	0.019	0.066	53.46	2.815	2.293	110.5	
Key point 4 (−2.61 Bohr): Δρ_H···*N*3_ = 0	O6H···O2	0.058	0.139	3.50	2.607	1.594	171.0	3.31
	N1H···N3	0.107	0.002	5.19	2.617	1.445	169.8	
	N2H···N4	0.022	0.075	21.37	2.801	2.169	118.3	
Key point 5 (−0.21 Bohr): Δρ_H···*N*4_ = 0	O6H···O2	0.050	0.129	3.84	2.662	1.657	170.8	3.53
	N1H···N3	0.061	0.100	7.18	2.691	1.670	158.7	
	N2H···N4	0.111	0.007	3.50	2.576	1.418	163.2	
Key point 6 (0.00 Bohr): TS${}_{\text{G}*\cdot C*\left(rWC\right)↔G{*}^{\prime }N2\cdot C\left(WC\right)}$	O6H···O2	0.049	0.131	3.84	2.663	1.662	170.6	4.55
	N1H···N3	0.059	0.105	7.29	2.692	1.682	158.2	
	N2-HN4	0.155	−0.214	3.17	2.575	1.287	164.5	
	N2H-N4	0.147	−0.161	3.53	2.575	1.312	164.5	
Key point 7 (0.02 Bohr): ρ_N2−H_ = ρ_H−N4_	O6H···O2	0.049	0.131	3.80	2.664	1.663	170.6	4.85
	N1H···N3	0.058	0.106	7.30	2.693	1.684	158.1	
	N2-H/H-N4	0.157	−0.225	3.50	2.575	1.286	164.7	
Key point 8 (0.26 Bohr): Δρ_N2···*H*_ = 0	O6H···O2	0.048	0.133	3.83	2.665	1.668	170.4	6.05
	N1H···N3	0.057	0.111	7.40	2.694	1.694	157.7	
	N2···HN4	0.108	0.005	3.21	2.580	1.419	165.0	
Key point 9 (4.12 Bohr): G${*}_{\text{N}2}^{{}^{\prime }}\cdot$C(rWC)	O6H···O2	0.037	0.117	3.87	2.750	1.768	170.4	8.85
	N1H···N3	0.033	0.095	8.50	2.876	1.921	153.8	
	N2···HN4	0.024	0.071	11.36	3.014	2.023	160.2	

*For footnote definitions see Table 3*.

**Table 6:** Electron-topological and structural characteristics of the specific intermolecular bonds revealed in the 9 key points and the polarity of the latters along the IRC of the IV. G*′·C*(H)↔G*_N7_·C(H) tautomerisation obtained at the B3LYP/6-311++G(d,p) level of theory in vacuum (see Figure 8).

**Complex**	**AH···B H-bond/ A-H/H-B covalent bond**	***ρ^a^***	***Δρ*^b^**	***100·ε^c^***	***${\text{d}}_{A\cdot \cdot \cdot B}^{\text{d}}$***	***${\text{d}}_{H\cdot \cdot \cdot B}^{\text{e}}$***	**_***∠AH*···*B***_*^**f**^***	**μ^g^**
Key point 1 (−6.04 Bohr): G*′·C*(H)	O6H···N4	0.047	0.102	4.88	2.710	1.742	160.7	5.16
	N7···HN3	0.032	0.101	5.68	2.834	1.791	180.0	
	C8H···O2	0.003	0.012	1.73	3.675	3.029	118.9	
Key point 2 (−1.17 Bohr):Δρ_O6···*H*_ = 0	O6H···N4	0.113	0.007	3.73	2.484	1.397	164.9	6.89
	N7···HN3	0.077	0.098	4.56	2.634	1.558	175.4	
	C8H···O2	0.006	0.022	15.15	3.344	2.716	116.8	
Key point 3 (−0.96 Bohr): ρ_O6−H_ = ρ_H−N4_	O6-H/H-N4	0.165	−0.262	1.52	2.428	1.214	166.3	8.27
	N7···HN3	0.080	0.089	4.45	2.631	1.544	175.6	
	C8H···O2	0.006	0.022	15.27	3.343	2.716	116.7	
Key point 4 (−0.74 Bohr): Δρ_H···*N*4_ = 0	O6···HN4	0.126	0.023	1.77	2.494	1.313	166.6	9.45
	N7···HN3	0.085	0.076	4.30	2.626	1.523	175.7	
	C8H···O2	0.006	0.022	14.99	3.338	2.710	116.8	
Key point 5 (−0.10 Bohr): Δρ_N7···*H*_ = 0	O6···HN4	0.091	0.136	2.15	2.534	1.432	167.7	9.96
	N7···HN3	0.111	0.004	3.72	2.589	1.420	175.7	
	C8H···O2	0.007	0.024	12.56	3.305	2.668	117.3	
Key point 6 (0.00 Bohr): TS${}_{\text{G}{*}^{\prime }\cdot C*\left(H\right)↔G*N7\cdot C\left(H\right)}$	O6···HN4	0.088	0.143	2.19	2.539	1.446	167.7	9.63
	N7-HN3	0.124	−0.053	3.48	2.583	1.374	176.0	
	N7H-N3	0.191	−0.493	3.58	2.583	1.210	176.0	
	C8H···O2	0.007	0.024	12.24	3.303	2.665	117.4	
Key point 7 (0.17 Bohr): ρ_N7−H_ = ρ_H−N3_	O6···HN4	0.084	0.152	2.23	2.543	1.463	167.7	8.84
	N7-H/H-N3	0.156	−0.231	3.09	2.580	1.288	176.2	
	C8H···O2	0.007	0.024	11.61	3.302	2.662	117.5	
Key point 8 (0.41 Bohr): Δρ_H···*N*3_ = 0	O6···HN4	0.079	0.161	2.28	2.548	1.482	167.6	7.73
	N7H···N3	0.112	0.006	4.55	2.583	1.414	176.3	
	C8H···O2	0.007	0.024	10.78	3.301	2.657	117.8	
Key point 9 (3.26 Bohr): G*_N7_·C(H)	O6···HN4	0.045	0.137	2.53	2.728	1.689	172.2	7.32
	N7H···N3	0.054	0.099	5.95	2.758	1.700	177.2	
	C8H···O2	0.006	0.022	16.54	3.324	2.711	115.8	

*For footnote definitions see Table 3*.

**Table 7:** Electron-topological and structural characteristics of the specific intermolecular bonds revealed in the 9 key points and the polarity of the latters along the IRC of the V. G*′·C*(rH)↔G*′_N7_·C(rH) tautomerisation obtained at the B3LYP/6-311++G(d,p) level of theory in vacuum (see Figure 10).

**Complex**	**AH···B H-bond/ A-H/H-B covalent bond**	***ρ^a^***	***Δρ*^b^**	***100·ε^c^***	***dA*···*B*^d^**	***dH*···*B*^e^**	**_***∠AH*···*B***_*^**f**^***	**μ^g^**
Key point 1 (−13.94 Bohr): G*′·C*(rH)	O6H···O2	0.034	0.120	2.25	2.733	1.785	160.6	5.43
	N7···HN3	0.042	0.101	2.73	2.838	1.797	176.3	
	C8H···N4	0.004	0.013	3.58	3.681	3.015	120.4	
Key point 2 (−1.33 Bohr): Δρ_N7···*H*_ = 0	O6H···O2	0.009	0.027	2.75	3.365	2.407	167.6	5.11
	N7···HN3	0.121	−0.007	3.60	2.545	1.377	167.2	
	C8H···N4	0.044	0.124	3.78	2.740	1.814	139.8	
Key point 3 (−1.12 Bohr): ρ_N67−H_ = ρ_H−N3_	O6H···O2	0.009	0.027	2.82	3.366	2.406	167.9	4.70
	N7-H/H-N3	0.161	−0.256	4.13	2.549	1.283	166.5	
	C8H···N4	0.047	0.124	4.09	2.730	1.783	141.6	
Key point 4 (−0.71 Bohr): Δρ_H···*N*3_ = 0	O6H···O2	0.009	0.027	2.77	3.367	2.405	168.1	4.47
	N7H···N3	0.110	0.003	4.75	2.564	1.430	163.8	
	C8H···N4	0.061	0.116	4.87	2.700	1.668	148.7	
Key point 5 (−0.20 Bohr): Δρ_H···*N*4_ = 0	O6H···O2	0.009	0.027	2.67	3.368	2.408	167.9	4.93
	N7H···N3	0.090	0.073	5.10	2.574	1.506	161.6	
	C8H···N4	0.111	−0.010	4.53	2.679	1.425	157.2	
Key point 6 (−0.04 Bohr): ρ_C8−H_ = ρ_H−N4_	O6H···O2	0.009	0.027	2.66	3.368	2.409	167.9	5.38
	N7H···N3	0.087	0.084	5.16	2.576	1.520	161.2	
	C8-H/H-N4	0.137	−0.202	2.32	2.677	1.341	158.5	
Key point 7 (0.00 Bohr): TS${}_{\text{G}{*}^{\prime }\cdot C*\left(rH\right)↔G{*}^{\prime }N7\cdot C\left(rH\right)}$	O6H···O2	0.009	0.027	2.66	3.368	2.409	167.9	5.51
	N7H···N3	0.086	0.087	5.18	2.576	1.524	161.1	
	C8-HN4	0.131	−0.174	2.36	2.677	1.320	158.8	
	C8H-N4	0.145	−0.169	4.22	2.677	1.404	158.8	
Key point 8 (0.36 Bohr): Δρ_C8···*H*_ = 0	O6H···O2	0.009	0.027	2.62	3.369	2.411	167.7	6.81
	N7H···N3	0.079	0.107	5.31	2.581	1.554	160.0	
	C8^−^···HN4	0.084	0.003	3.08	2.681	1.581	160.7	
Key point 9 (9.18 Bohr): G*′_N7_·C(rH)	O6H···O2	0.022	0.080	3.15	2.918	1.968	162.9	9.01
	N7H···N3	0.049	0.114	6.60	2.758	1.743	167.8	
	C8^−^···HN4	0.016	0.038	6.19	3.302	2.316	160.1	

*For footnote definitions see Table 3*.

**Table 8:** Patterns of the specific intermolecular interactions including AH···B H-bonds and loosened A-H-B covalent bridges that sequentially replace each other along the IRC of the investigated tautomerisations *via* the SPT or DPT obtained at the B3LYP/6-311++G(d,p) level of theory (see Figure 12).

**Patterns**	**IRC range, Bohr**	**Specific intermolecular interactions, forming patterns**
I. G*·C*(rWC)↔G^+^·C^**−**^(rWC)↔G·C*_O2_(rWC)		
I	[−4.49 ÷−0.27)	(G)O6H···O2(C), (G)N1···HN3(C), (G)N2H···N4(C)
II	[−0.27 ÷ 0.12)	(G)O6H···O2(C), (G)N1-H/H-N3(C), (G)N2H···N4(C)
III	[0.12 ÷ 2.13)	(G)O6H···O2(C), (G)N1H···N3(C), (G)N2H···N4(C)
IV	[2.13 ÷ 2.51)	(G)O6-H/H-O2(C), (G)N1H···N3(C), (G)N2H···N4(C)
V	[2.51 ÷ 4.75]	(G)O6···HO2(C), (G)N1H···N3(C), (G)N2H···N4(C)
II. G*·C*(rWC)↔G^+^·C^**−**^(rWC)↔G*_N2_·C(rWC)		
I	[−4.49÷−0.27)	(G)O6H···O2(C), (G)N1···HN3(C), (G)N2H···N4(C)
II	[−0.27 ÷ 0.12)	(G)O6H···O2(C), (G)N1-H/H-N3(C), (G)N2H···N4(C)
III	[0.12 ÷ 4.52)	(G)O6H···O2(C), (G)N1H···N3(C), (G)N2H···N4(C)
IV	[4.52 ÷ 4.91)	(G)O6H···O2(C), (G)N1H···N3(C), (G)N2-H/H-N4(C)
V	[4.91 ÷ 6.05]	(G)O6H···O2(C), (G)N1H···N3(C), (G)N2···HN4(C)
III. G*·C*(rWC)↔G*′_N2_·**C(rw**_**WC**_**)**		
I	[−10.37 ÷−4.79)	(G)O6H···O2(C), (G)N1···HN3(C), (G)N2H···N4(C)
II	[−4.79 ÷−3.23)	(G)O6H···O2(C), (G)N1···HN3(C), (G)N2···N4(C)
III	[−3.23 ÷−2.71)	(G)O6H···O2(C), (G)N1-H/H-N3(C), (G)N2H···N4(C)
IV	[−2.71 ÷−0.16)	(G)O6H···O2(C), (G)N1H···N3(C), (G)N2H···N4(C)
V	[−0.16 ÷ 0.21)	(G)O6H···O2(C), (G)N1H···N3(C), (G)N2-H/H-N4(C)
VI	[0.21 ÷ 4.12]	(G)O6H···O2(C), (G)N1H···N3(C), (G)N2···HN4(C)
IV. G*′·C*(H)↔G*_N7_·C(H)		
I	[−6.04 ÷−1.17)	(G)O6H···N4(C), (G)N7···HN3(C), (G)C8H···O2(C)
II	[−1.17 ÷−0.74)	(G)O6-H/H-N4(C), (G)N7···HN3(C), (G)C8H···O2(C)
III	[−0.74 ÷−0.10)	(G)O6···HN4(C), (G)N7···HN3(C), (G)C8H···O2(C)
IV	[−0.10 ÷ 0.37)	(G)O6···HN4(C), (G)N7-H/H-N3(C), (G)C8H···O2(C)
V	[0.37 ÷ 3.26]	(G)O6···HN4(C), (G)N7H···N3(C), (G)C8H···O2(C)
V. G*′·C*(rH)↔G*′_N7_·C(rH)		
I	[−13.94 ÷−1.33)	(G)O6H···O2(C), (G)N7···HN3(C), (G)C8H···N4(C)
II	[−1.33 ÷−0.71)	(G)O6H···O2(C), (G)N7-H/H-N3(C), (G)C8H···N4(C)
III	[−0.71 ÷−0.20)	(G)O6H···O2(C), (G)N7H···N3(C), (G)C8H···N4(C)
IV	[−0.20 ÷ 0.31)	(G)O6H···O2(C), (G)N7H···N3(C), (G)C8-H/H-N4(C)
V	[0.31 ÷ 9.18]	(G)O6H···O2(C), (G)N7H···N3(C), (G)C8···HN4(C)

### Tautomerisation of the Reverse Löwdin G^*^·C^*^(rWC) Base Pair *via* the SPT: I. G^*^·C^*^(rWC)↔G^+^·C^−^(rWC)↔G·${\text{C}}_{\text{O2}}^{*}$(rWC) and II. G^*^·C^*^(rWC)↔G^+^·C^−^(rWC)↔${\text{G}}_{\text{N2}}^{*}\cdot$C(rWC)

For the first time we have discovered three local minima on the hypersurface of the electronic energy of the G^*^·C^*^(rWC) base pair corresponding to the high-energy tautomerised G·${\text{C}}_{\text{O}2}^{*}$(rWC), G^+^·C^−^(rWC), and ${\text{G}}_{\text{N}2}^{*}\cdot$C(rWC) base pairs (Figure 1, Table 1). All of them are stabilized by the participation of three intermolecular H-bonds, among which the upper O6H…O2/O2H…O6 H-bonds are the strongest (Table 2).

In fact, the tautomerisation of the G^*^·C^*^(rWC) base pair with relative Gibbs free energy ΔG = 0.00 kcal·mol^−1^ starts from the single transfer of the proton localized at the N3 nitrogen atom of the C base to the N1 nitrogen atom of the G base along the intermolecular H-bond (C)N3H…N1(G). This G^*^·C^*^(rWC)↔G^+^·C^−^(rWC) tautomerization process occurs *via* the TS_G*·C*(rWC)↔G+·C^−^(rWC)_ (C_s_ symmetry) (ΔG = 4.38 kcal·mol^−1^) containing N1-H-N3 covalent bridge and further proceeds through the intermediate—tight ion pair G^+^·C^−^(rWC) (ΔG = 4.44 kcal·mol^−1^) (C_s_ symmetry), which is the point of bifurcation. By the way, it should be noted that this is the first case of the reliable fixation of the ionic pair of bases, formed as a result of the SPT along the intermediate molecular H-bond, which is involved in its stabilization. Similar attempts to localize such structures for the A·T(WC) and G·C(WC) DNA base pairs didn't lead to result.

Further the G^*^·C^*^(rWC)↔G^+^·C^−^(rWC) tautomerisation reaction proceeds according two scenarios *via* the proton transfer along:

- the (G)O6H….O2(C) H-bond through the TS_G^+^·C^−^(rWC)↔G·C*O2(rWC)_ (ΔG = 3.64 kcal·mol^−1^; C_s_ symmetry) with O6-H-O2 covalent bridge leading to the G·${\text{C}}_{\text{O}2}^{*}$(rWC) product (C_s_ symmetry);
- the (G)N2H….N4(C) H-bond through the TS_G^+^·C^−^(rWC)↔G*N2·C(rWC)_ (ΔG = 9.27 kcal·mol^−1^; C_s_ symmetry) N2-H-N4 covalent bridge leading to the ${\text{G}}_{\text{N}2}^{*}$·C(rWC) product (C_s_ symmetry).

It attracts attention that electronic ΔE_int_ and Gibbs free ΔG_int_ energies of the interaction for the tautomerised G·${\text{C}}_{\text{O}2}^{*}$(rWC) (ΔE_int_ = −39.67/ΔG_int_ = −26.53) and ${\text{G}}_{\text{N}2}^{*}$·C(rWC) (ΔE_int_ = −41.10/ΔG_int_ = −26.10) base mispairs exceed the values for the initial G^*^·C^*^(rWC) base mispair (ΔE_int_ = −20.21/ΔG_int_ = −7.40) and also canonical G·C(WC) base pair (ΔE_int_ = −29.28/ΔG_int_ = −15.97 kcal·mol^−1^) (Brovarets' and Hovorun, 2014a).

Moreover, it was revealed that all three formed high-energy complexes—the G^+^·C^−^(rWC) ion pair and G·${\text{C}}_{\text{O}2}^{*}$(rWC), ${\text{G}}_{\text{N}2}^{*}$·C(rWC) tautomers of the Löwdin G^*^·C^*^(rWC) base pair are dynamically unstable structures, since for them the zero energy of the vibrations, which frequency become imaginary at the TS of tautomerisation, significantly exceeds the reverse electronic barrier (363.8, 668.1, and 454.7 cm^−1^) (Table 1). Moreover, Gibbs free energies of the reverse barrier for the G^*^·C^*^(rWC)↔G^+^·C^−^(rWC) and G^+^·C^−^(rWC)↔${\text{G}}_{\text{N}2}^{*}$·C(rWC) tautomeric transformations are negative (−0.06 and −1.12 kcal·mol^−1^, accordingly) and for the G^+^·C^−^(rWC)↔G·${\text{C}}_{\text{O}2}^{*}$(rWC)—it is less (0.13 kcal·mol^−1^) than *kT* (0.62 kcal·mol^−1^ under normal conditions) (Table 1).

So, in fact the Löwdin G^*^·C^*^(rWC) base pair does not tautomerise to the novel G·${\text{C}}_{\text{O}2}^{*}$(rWC) and ${\text{G}}_{\text{N}2}^{*}$·C(rWC) base mispairs *via* the SPT along the intermolecular H-bonds. However, despite this verdict, obtained data can be useful as an analogy or even as a heuristic push at the investigation of the tautomerisation mechanisms of the H-bonded complexes of any nature.

### Tautomerisation of the Reverse Löwdin G^*^·C^*^(rWC) Base Pair *via* the DPT: III. G^*^·C^*^(rWC)↔G*′_N2_·C(rWC)

We have also detected the unusual tautomerisation of the reverse Lowdin's G^*^·C^*^(rWC) DNA base mispair *via* the asynchronous [with a level of asynchrony 3.49 Bohr (Brovarets' and Hovorun, 2019b)] concerted DPT to the G*′_N2_·C(rWC) DNA base mispair with *trans-*oriented N2H imino group of the G DNA base, in which participates the protons at the N3(C) and N2(G) nitrogen atoms moving in opposite directions. Unusual nature of this process consists in the fact that the transitions of the protons from N3(C) to N1(G) and from N2(G) to N4(C) along the intermolecular H-bonds provokes the rotation of the NH_2_ amino group of the G base into the *trans*-position relatively the neighboring double C2N3(G) bond. As a results, this G^*^·C^*^(rWC)↔G*′_N2_·C(rWC) tautomerisation reaction proceeds through the substantially non-planar intermediate – G^+′^_N2_·C^−^(rWC) ion pair with non-planar NH_2_ amino group (C_1_ symmetry), the substantially non-planar TS_G*·C*(rWC)↔G*′_N2_·C(rWC)_ (C_1_ symmetry), and substantially non-planar product of this reaction—the G*′_N2_·C(rWC) DNA base mispair (C_1_ symmetry). This DNA base mispair is stabilized by the participation of the three intermolecular H-bonds—(G)O6H…O2(C), (G)N1H…N3(C), and (C)N4H…N2(G) and represents itself quite stable structure (ΔE_int_ = −33.41/ΔG_int_ = −18.99 kcal·mol^−1^). Its characteristic structural feature is the substantial non-flatness (∠N1C6C2N3 = 23.3°; ∠C2N2N4C4 = 53.8°) and the exit from the plane of the purine ring of the O6H (∠HO6C6N1 = 10.8°), N1H (∠HN1C2N3 = -157.4°), and N2H (∠HN2C2N3 = -167.6°) external groups with the *trans-*orientation relative to the neighboring bond C2N3.

Moreover, we have revealed that the ${\text{G}}_{\text{N}2}^{*}\cdot$C(rWC) and G*′_N2_·C(rWC) DNA base pairs interconvert *via* the conformational rotation of the N2H imino group around the C2N2 bond through the Gibbs freee energy barrier 31.65 kcal·mol^−1^ (Figure 1, Tables 1–2).

### Tautomerisation of the Hoogsteen G^*^′·C^*^(H) Base Pair *via* the Classical DPT: IV. G^*^′·C^*^(H)↔${\text{G}}_{\text{N7}}^{*}\cdot$C(H)

The tautomerisation of the Hoogsteen G^*^′·C^*^(H) base pair (ΔG = 0.00 kcal·mol^−1^; C_s_ symmetry) is possible *via* one-single way—through the asynchronous DPT (the values of the asynchronity consists 1.58 Bohr) (for more details see further discussion and Figure 8) along two intermolecular antiparallel lower (G)O6H…N4(C) and (C)N3H…N7(G) H-bonds through the TS_G*′·C*(H)↔G*N7·C(H)_ (ΔG = 4.01 kcal·mol^−1^; C_s_ symmetry) connected by the N7-H-N3 covalent bridge with further formation of the dynamically-unstable ${\text{G}}_{\text{N}7}^{*}\cdot$C(H) base mispair with small lifetime τ = 4.46·10^−13^ s (ΔG = 3.20 kcal·mol^−1^; C_s_ symmetry). The G^*^′·C^*^(H)↔${\text{G}}_{\text{N}7}^{*}\cdot$C(H) tautomerisation starts from the initial transfer of the hydrogen atom localized at the O6 oxygen atom of the G^*^′ DNA base to the N4 nitrogen atom of the C^*^ DNA base within the G^*^′·C^*^(H) DNA base pair and then through the G^+^·C^−^(H) Hoogsteen base pair *via* the proton transfer from the N3 nitrogen atom to the N7 nitrogen atom leading to the formation of the ${\text{G}}_{\text{N}7}^{*}\cdot$C(H) DNA base mispair by the participation of the rare ${\text{G}}_{\text{N}7}^{*}$ tautomer and canonical C DNA base.

Notably, that initial and final structures involved in this tautomerisation process are stabilized by the participation of three intermolecular H-bonds (Table 2), one of which (G)C8H…O2(C) is non-standard with energy 1.15 kcal·mol^−1^.

Notably, electronic (ΔE_int_ = −35.66) and Gibbs free (Δ*G*_int_ = −22.05 kcal·mol^−1^) energies of the interaction for the terminal ${\text{G}}_{\text{N}7}^{*}\cdot$C(H) base mispair exceed the values for the initial base mispair (ΔE_int_ = −21.24/ΔG_int_ = −8.91 kcal·mol^−1^). At this, total energies of the H-bonds make a great contribution to the electronic interaction energy−78.1% for the G^*^′·C^*^(H) DNA base mispair and 51.7% for the ${\text{G}}_{\text{N}7}^{*}\cdot$C(H) DNA base mispair.

All low-frequency intermolecular vibrations of the ${\text{G}}_{\text{N}7}^{*}\cdot$C(H) base mispair, which periods are in the range 8.06·10^−13^-1.16·10^−12^ s, can't develop during its lifetime. This situation is typical for the structures, which are deprived of dynamic stability (Brovarets' and Hovorun, 2019b).

So, in this case in fact the G^*^′·C^*^(H) base pair does not tautomerise *via* the DPT similarly to the previous G^*^·C^*^(rWC) base pair.

### Tautomerisation of the Reverse Hoogsteen G^*^′·C^*^(rH) Base Pair *via* the DPT by the Participation of the C8H(G^*^′) Group: V. G^*^′·C^*^(rH)↔G^*′^_N7_·C(rH)

The G^*^′·C^*^(rH) base pair differs from two previous ones, since it tautomerises *via* the asynchronous DPT (with the value of asynchronity 1.69 Bohr) along the intermolecular antiparallel (C)N3H…N7(G) and (G)C8H…N4(C) H-bonds with further formation of the yilidic form G*′_N7_ of the G DNA base (Govorun et al., 1995a,b; Kondratyuk et al., 2000), which is characterized by the transferred proton of the C8H group to the neighboring N7 nitrogen atom. The G^*^′·C^*^(rH)↔G*′_N7_·C(rH) tautomerisation proceeds *via* the initial transfer of the proton localized at the N3 nitrogen atom of C base to the N7 nitrogen atom of G base through the formation of the G^+′^_N7_·C^−^ ion pair followed by further proton transfer localized at the C8 carbon atom of G^*^′ base to the N4 nitrogen atom of C^*^ base. Notably, that TS of this process—TS_G*′·C*(rH)↔G*′_N7_·C(rH)_–has planar structure (C_s_ symmetry) and contains C8-H-N4 covalent bridge, which angle is 158.8°.

This process is become possible due to the fact that G base, from one side, is CH-acid (Kondratyuk et al., 2000) and from the other—it is able to transfer into the zwitterionic tautomer—so-called yilidic form (Govorun et al., 1995a,b; Kondratyuk et al., 2000). Analysis of the obtained data (Table 1) evidences that G*′_N7_·C(rH) tautomer is dynamically stable structure with quite long lifetime (τ = 5.15·10^−12^ s). Characteristically, that all 6 low-frequency intermolecular vibrations of the G*′_N7_·C(rH) base mispair, which period are in the range 6.83·10^−13^-1.51·10^−12^ s, can develop during this lifetime.

This is the first case (Brovarets' et al., 2018f), when the product of the tautomerization of the H-bonded base pair by the participation of the yilidic purine base is dynamically stable structure. However, formed G*′_N7_·C(rH) base pair has low population−2.3·10^−17^ under normal conditions, which complicates the understanding of its biological role.

At the same time, such structures represent a considerable interest from a theoretical point of view, in particular, they contain unusual (C)N4H…C8^−^(G) H-bond (Table 2), which supplements existing data about the nature of the H-bonding in the pairs of DNA/RNA bases (Brovarets' et al., 2014e).

### Profiles of the Physico-Chemical Parameters of the Investigated SPT and DPT Tautomerisations

We have investigated in details the mechanisms of the abovementioned processes of the tautomerisation of the reverse Lowdin's G^*^·C^*^(rWC), Hoogsteen G^*^′·C^*^(H), and reverse Hoogsteen G^*^′·C^*^(rH) base pairs *via* the PT along the intermolecular H-bonds. Tautomerisations proceed in *a synchronous concerted manner via the stepwise SPT* in the case of the I. G^*^·C^*^(rWC)↔G^+^·C^−^(rWC)↔G·${\text{C}}_{\text{O}2}^{*}$(rWC) and II. G^*^·C^*^(rWC)↔G^+^·C^−^(rWC)↔${\text{G}}_{\text{N}2}^{*}\cdot$C(rWC) reactions, while in *an asynchronous concerted manner via the DPT* in the case of the III. G^*^·C^*^(rWC)↔G*′_N2_·C(rWC), IV. G^*^′·C^*^(H)↔${\text{G}}_{\text{N}7}^{*}$·C(H) and V. G^*^′·C^*^(rH)↔G*′_N7_·C(rH) reactions (Figures 2, 4, 6, 8, 10, Tables 3–7).

We have established following regularities of the general character for the obtained sweeps of the most important physico-chemical parameters along the IRC.

The widths of the reaction zone of the investigated reactions starting from the reagent and till the product are almost the same for the I. G^*^·C^*^(rWC)↔G·${\text{C}}_{\text{O}2}^{*}$(rWC) (9.24); II. G^*^·C^*^(rWC)↔${\text{G}}_{\text{N}2}^{*}$·C(rWC) (10.54) and IV. G^*^′·C^*^(H)↔${\text{G}}_{\text{N}7}^{*}$·C(H) (9.30 Bohr), while these widths are significantly larger for the III. G^*^·C^*^(rWC)↔G*′_N2_·C(rWC) (14.49) and V. G^*^′·C^*^(rH)↔G*′_N7_·C(rH) (23.12 Bohr) reactions (Figures 2, 4, 6, 8, 10).

All tautomerisation processes are dipole-active, since they are followed by the significant change of the dipole moment μ of the tautomerising base pair (Figures 3C, 5C, 7C, 9C, 11C). These dependencies are U-like with maximal and minimal values located in the transition state zone for the I. G^*^·C^*^(rWC)↔G·${\text{C}}_{\text{O}2}^{*}$(rWC) SPT (values of μ change in the range: 3.15–6.54); II. G^*^·C^*^(rWC)↔${\text{G}}_{\text{N}2}^{*}$·C(rWC) SPT (3.15–7.15); III. G^*^·C^*^(rWC)↔G*′_N2_·C(rWC) DPT (2.81–8.84) and V. G^*^′·C^*^(rH)↔G*′_N7_·C(rH) DPT (4.47–9.01 D) reactions, while it is reverse with maximal values at the transition state zone for the IV. G^*^′·C^*^(H)↔${\text{G}}_{\text{N}7}^{*}$·C(H) reaction (5.16–10.12 D) DPT (Figures 3C, 5C, 7C, 9C, 11C).

Tautomerisation process does not change the configuration of the base pair—complexes slightly compress on several dozens of Angstrom at the zone of the TSs of tautomerisation. TSs demonstrate significant deviations from a plane in the case, when steric conflicts arise between the interacting exocyclic groups (Figures 2, 4, 6, 8, 10).

According to the authors' conception (Brovarets' and Hovorun, 2013a,b,c,d, 2014c,d, 2015h,i; Brovarets' et al., 2013a,b, 2014a,b,c, 2015), it was introduced key points, namely 11 key points were obtained in the cases of the I. G^*^·C^*^(rWC)↔G·${\text{C}}_{\text{O}2}^{*}$(rWC) and II. G^*^·C^*^(rWC)↔${\text{G}}_{\text{N}2}^{*}$·C(rWC) SPT along the IRC in contrast to the processes of the III. G^*^·C^*^(rWC)↔G*′_N2_·C(rWC), IV. G^*^′·C^*^(H)↔${\text{G}}_{\text{N}7}^{*}$·C(H) and V. G^*^′·C^*^(rH)↔G*′_N7_·C(rH) DPT, for which 9 key points have been localized.

At this, it was obtained typical crossings of the profiles for the electron density ρ, the Laplacian of the electron density Δρ and the distance d_AH/HB_ between the hydrogen and electronegative A or B atoms for the H-bonds involved in the tautomerisation, notifying the equalization of these parameters. They occur at the 3rd and 9th key points in the case of the reaction I, 3rd and 8th—for the reaction II, 3rd and 7th—for the reactions III and IV and 3rd and 6th—for the reaction V (Figures 3G,H,K, 5G,H,K, 7G,H,K, 9G,H,K, 11G,H,K).

One and the same regularity is observed for the dE/dIRC function in all cases of tautomerisations—with two local maxima and two local minima achieved at the TS zone (Figures 3B, 5B, 7B, 9B, 11B).

Also it was observed five patterns for the energy E_HB_ of the intermolecular H-bonds, estimated by the EML method (Espinosa et al., 1998; Matta et al., 2006b; Mata et al., 2011; Lecomte et al., 2015; Alkorta et al., 2016, 2017), along the IRC for the I, II, IV and V reactions and four patterns—for the III reaction (Figure 12, Table 8). These sweeps allow to estimate numerically the cooperativity of the neighboring H-bonds according to the methodology, proposed by us earlier (Brovarets' and Hovorun, 2019b). It was established the general pattern—the anti-parallel H-bonds amplify each other and parallel—weaken each other (Turaeva and Brown-Kennerly, 2015). Moreover, some of the dependencies of the energy E_HB_ of the intermolecular H-bonds exist during entire IRC, such as (G)N2H…N4(C) for the I. G^*^·C^*^(rWC)↔G·${\text{C}}_{\text{O}2}^{*}$(rWC) reaction, (G)O6H…O2(C) for the II. G^*^·C^*^(rWC)↔${\text{G}}_{\text{N}2}^{*}$·C(rWC) reaction, (G)O6H…O2(C) for the III. G^*^·C^*^(rWC)↔G*′_N2_·C(rw_WC_) reaction, (G)C8H…O2(C) (its energy remains almost unchangeable during the IRC) for the G^*^′·C^*^(H)↔${\text{G}}_{\text{N}7}^{*}$·C(H) reaction and (G)O6H…O2(C) for the G^*^′·C^*^(rH)↔G*′_N7_·C(rH) reaction. In the case of the III. G^*^·C^*^(rWC)↔G*′_N2_·C(rw_WC_) reaction, some of the H-bonds transform into the van der Waals contact and then to another H-bonds during the IRC: (G)N2H…N4(C) H-bond → (G)N2…N4(C) vdW contact → (G)N2H…N4(C) H-bond for the III. G^*^·C^*^(rWC)↔G*′_N2_·C(rw_WC_) reaction (Figure 12). For more details according the patterns refer to Table 8.

Finally, we would like to note some general regularities, which are characteristic for all without exclusion processes of tautomerisation.

Thus, in the vast majority of cases base pairs are plane symmetric structures during the entire PT and DPT tautomerization processes along the IRC, despite the ability of the DNA bases for the out-of-plane bending (Govorun et al., 1992; Hovorun et al., 1999; Nikolaienko et al., 2011), excluding two mentioned above cases, when there are deviation from the planarity—III. G^*^·C^*^(rWC)↔G*′_N2_·C(rWC) (Figure 1).

Interestingly, the total energy of the intermolecular H-bonds only partially contributes to the electron energy of the monomers interactions among all without any exceptions H-bonded structures investigated in this work (see Figures 1, 12). This result is in a good accordance with generalized literature data (Brovarets' and Hovorun, 2014e, 2019b).

## Conclusions

For the first time the tautomerisation of the biologically-important conformers of the G^*^·C^*^ DNA base pair - reverse Löwdin G^*^·C^*^(rWC), Hoogsteen G^*^′·C^*^(H) and reverse Hoogsteen G^*^′·C^*^(rH) pairs - was investigated and thoroughly analyzed. It was found out that the G^*^·C^*^(rWC)↔G^+^·C^−^(rWC)↔G·${\text{C}}_{\text{O}2}^{*}$(rWC) and G^*^·C^*^(rWC)↔G^+^·C^−^(rWC)↔${\text{G}}_{\text{N}2}^{*}\cdot$C(rWC) tautomerization processes occur *via* the two-stage sequential SPT *via* dynamically-unstable zwitterion-like G^+^·C^−^(rWC) intermediate, while the G^*^·C^*^(rWC)↔G*′_N2_·C(rw_WC_), G^*^′·C^*^(H)↔${\text{G}}_{\text{N}7}^{*}$·C(H), and G^*^′·C^*^(rH)↔G*′_N7_·C(rH) tautomerization processes occur through the one-stage DPT. At this, proton transfer along the non-canonical (G^*^)CH…N(C^*^) H-bond is accompanied by the significant deviation of the C-H-N bridge at the TS.

Obtained data evidence that among the G^*^·C^*^(rWC), G′·C^*^(H) and G^*^′·C^*^(rH) base pairs only the tautomerisation of the latest of them lead to the formation of the dynamically stable G*′_N7_·C(rH) base pair with lifetime 5.15 ps with a miserable population 2.3·10^−17^.

Moreover, it was revealed that the I. G^*^·C^*^(rWC)↔G^+^·C^−^(rWC)↔G·${\text{C}}_{\text{O}2}^{*}$(rWC) and II. G^*^·C^*^(rWC)↔G^+^·C^−^(rWC)↔${\text{G}}_{\text{N}2}^{*}\cdot$C(rWC) tautomerization reactions proceed in *a synchronous concerted manner via the stepwise SPT*, while the III. G^*^·C^*^(rWC)↔G*′_N2_·C(rWC), IV. G^*^′·C^*^(H)↔${\text{G}}_{\text{N}7}^{*}\cdot$C(H), and V. G^*^′·C^*^(rH)↔G*′_N7_·C(rH) reactions occur in *an asynchronous concerted manner via the DPT*.

We have also established dependencies of the most important physico-chemical parameters along the IRC enabling to understand more precisely the inherent nature of the investigated processes.

## Data Availability

The datasets generated for this study are available on request to the corresponding author.

## Author Contributions

OB analysis and preparation of the current literature survey, discussion of the strategy of the current investigation, study conception and design, acquisition of data, drafting of manuscript analysis and interpretation of data, performance of calculations, discussion of the obtained data, preparation of the numerical data for Tables 1–8, graphical materials for Figures 1–12, and text of the manuscript. TO preparation of the numerical data for Tables 1–8 and graphical materials for Figures 1–12. DH study conception, critical revision of manuscript, proposition of the task of the investigation, discussion of the obtained data, and preparation of the text of the manuscript. All authors were involved in the proofreading of the final version of the manuscript.

### Conflict of Interest Statement

The authors declare that the research was conducted in the absence of any commercial or financial relationships that could be construed as a potential conflict of interest.
